# Impaired autophagic activity and ATG4B deficiency are associated with increased endoplasmic reticulum stress-induced lung injury.

**DOI:** 10.18632/aging.101532

**Published:** 2018-08-27

**Authors:** Mariana Maciel, Daniel Hernández-Barrientos, Iliana Herrera, Moisés Selman, Annie Pardo, Sandra Cabrera

**Affiliations:** 1 Department of Cell Biology, Facultad de Ciencias, Universidad Nacional Autónoma de México, Mexico City, Mexico; 2 Instituto Nacional de Enfermedades Respiratorias “Ismael Cosío Villegas”, Mexico City, México

**Keywords:** lung fibrosis, aging, ER stress, autophagy, ATG4B, epithelial apoptosis

## Abstract

Aging is the main risk factor for the development of idiopathic pulmonary fibrosis (IPF), a progressive and usually lethal lung disorder. Although the pathogenic mechanisms are uncertain, endoplasmic reticulum (ER) stress and impaired proteostasis that have been linked with aging are strongly associated with the pathogenesis of IPF. Using the Atg4b-deficient mice as a model, that partially reproduces the autophagy deficient conditions reported in aging and IPF lungs, we show for the first time how autophagy impairment and ER stress induction, contribute simultaneously to development of lung fibrosis in vivo. Increased expression of ER stress markers, inflammation and apoptosis of alveolar epithelial cells were observed in Atg4b-deficient mice compared to WT mice, when treated with the ER stress inducer tunicamycin. After tunicamycin treatment, Atg4b null lungs showed accumulation of its substrate LC3-I, demonstrating that these mice failed to induce autophagy despite the ER stress conditions. We also showed that compromised autophagy in lungs from Atg4b null mice is associated with exacerbated lung damage, epithelial apoptosis and the development of lung fibrosis at 21 days after tunicamycin treatment. Our findings indicate that ATG4B protein and autophagy are essential to mitigate ER stress and to prevent tunicamycin-induced epithelial apoptosis and lung fibrosis.

## Introduction

Idiopathic Pulmonary Fibrosis (IPF) is a chronic and progressive aging-associated disease of unknown etiology. IPF usually affects individuals over 60 years old and its prevalence increases considerably with age [1]. The disease is characterized by an aberrant activation of alveolar epithelial cells and accumulation of fibroblasts and myofibroblasts along with excessive production of extracellular matrix resulting in the abnormal scarring of the lung parenchyma [1,2]. The linkage of aging with this disorder is uncertain, but a number of changes associated with aging, including defective autophagy, endoplasmic reticulum (ER) stress, telomere attrition, altered proteostasis, mitochondrial dysfunction and cell senescence have been revealed in IPF lungs [2,3].

One of the hallmarks of aging is the loss of proteostasis characterized by perturbations in pathways involved in protein synthesis, folding, trafficking, and secretion, with impaired activity of the intracellular proteolytic systems such autophagy and proteasome. Loss of proteostasis could result in accumulation and aggregation of misfolded proteins leading to ER stress and activation of the unfolded protein response (UPR) [4,5]. Importantly, ER stress biomarkers has been found elevated mainly in epithelial cells from lungs of patients with familial IPF (primarily associated with mutations in the surfactant protein C (*SFTPC*) gene), but also in sporadic IPF [6,7]. Evidence from in vitro studies in cultured type II alveolar epithelial cells (AEC II), have revealed that expression of L188Q *SFTPC* mutant results in a precursor protein that cannot be folded properly in the ER, leading to ER stress and activation of UPR [8,9]. Moreover, conditional expression of mutant L188Q *SFTPC* in AEC II or tunicamycin treatment in mouse lung, induces ER stress characterized by an increased expression of BiP and XBP1 splicing, and exacerbates epithelial apoptosis and lung remodeling in bleomycin-induced lung fibrosis [6,7]. Together, these studies strongly support the involvement of ER stress in the development of lung fibrosis.

It is well known that ER stress induces autophagy, targeting misfolded proteins to degradation and promoting cell survival [10,11]. However, lung tissues from IPF patients demonstrate evidence of decreased autophagic activity despite activation of pathways known to promote autophagy, such as ER stress, increased HIF1α level, oxidative stress or mitochondrial dysfunction [12,13]. Moreover, we have shown that mice deficient in the cysteine-protease ATG4B, which exhibit a decrease of basal and induced autophagy, are more susceptible to bleomycin-induced lung injury and fibrosis, upholding the protective role for autophagy activity and ATG4B in the development of lung fibrosis [14,15]. ATG4B activity is essential for an appropriate autophagic activity in mammals, enabling the correct activation and localization of LC3, autophagosome biogenesis and maturation, but also to maintain a balance between lipidated and unlipidated forms of LC3 and its recycling when the autophagy flux is increased and autophagic activity is enhanced [14,16–18]. The relationship between ER stress and autophagy in the pathogenesis of IPF has not been elucidated. In this study, we used our *Atg4b* null mice, which displays systemic reduced autophagy, as a model that could mimic the impaired autophagic activity observed in aging and in the lungs of patients with IPF, to explore in vivo the role of autophagy in response to ER stress induced lung injury and fibrosis. We provide evidence that tunicamycin-induced ER stress and lung injury is exacerbated when autophagy is compromised. In lungs from *Atg4b^-/-^* mice, tunicamycin treatment leads to activation of UPR response, increased inflammation and epithelial apoptosis compared to WT littermates. At 3 and 21 days post-tunicamycin administration, the severity of lung injury characterized by thickness of alveolar septa and inflammatory cell infiltrate was markedly more severe in *Atg4b* null mice. Our data indicate that ATG4B and autophagic response have a cytoprotective effect against ER stress in lung and prevents tissue injury.

## RESULTS

### Tunicamycin treatment activates autophagy and ATG4B expression in mouse lung epithelial cells

**Figure 1:**
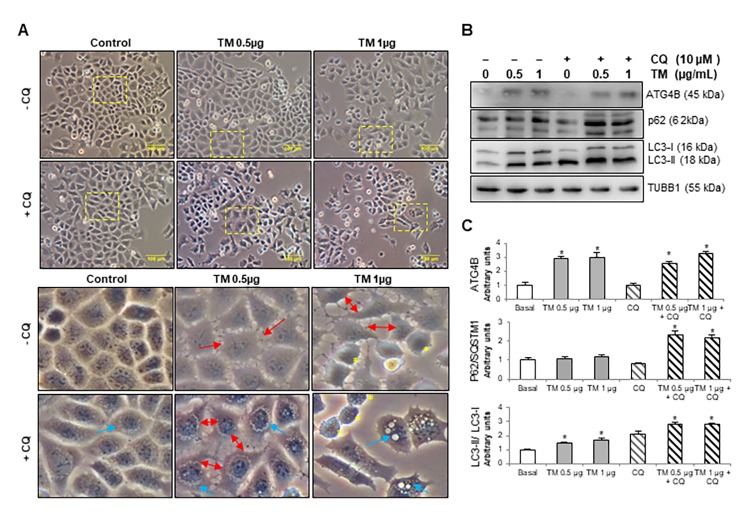
**Tunicamycin induces changes in cell morphology, ATG4B expression and autophagy activation in MLE12 cells**. (**A**) Phase contrast images of mouse epithelial MLE12 cells treated with vehicle control or 0.5 and 1 μg/ml tunicamycin alone or in combination with chloroquine for 24h. The line scale bar represents 100 μm. Lower panels represent magnified pictures of boxed area in the corresponding upper panels. Red arrows point out los**s** of cell-cell contacts, blue arrows indicate accumulation of vacuoles in cytoplasm and yellow asterisk point out apoptotic-like cells. (**B**) Representative immunoblots of ATG4B, LC3-I/ II, and p62 in mouse alveolar epithelial MLE12 cells treated with vehicle control or 0.5 and 1 μg/ml tunicamycin alone or in combination with chloroquine for 24h. β-tubulin was used as loading control. (**C**) Densitometry. Protein levels were normalized to vehicle control. Results represent mean ± SD. Statistical significance was determined by one-way ANOVA (* p< 0.05).

Numerous studies have demonstrated that alveolar epithelial cell (AEC) dysfunction and apoptosis have an initial key role in the pathogenesis of IPF [8,19,20]. To investigate whether autophagy is induced in AEC during ER stress and if autophagy provides cytoprotection, we evaluated the autophagic activity in MLE12 mouse alveolar epithelial cells after treatment with tunicamycin, a potent inductor of ER stress that inhibits N-linked protein glycosylation. Cells were incubated in presence or absence of the autophagy inhibitor chloroquine and treated with 0.5 and 1 μg/ml tunicamycin for 24 h. As shown in Figure 1A, by using phase contrast microscopy, we observed that tunicamycin induced changes in cell morphology and while vehicle-treated control MLE12 cells have the typical cuboidal morphology, tunicamycin-treated cells lost their cuboidal shape, and cell-cell contacts, and developed an elongated shape with cytoplasmic extensions (Fig.1A, red arrows in insets). Chloroquine treatment leads to membrane-enclosed vacuoles formation in control MLE12, and combination of tunicamycin + chloroquine induced an accumulation of vacuoles and dilation of autolysosomes (Fig.1A, blue arrows in insets). The turnover of the autophagosomal markers LC3B-II and p62, and ATGB4 levels were examined by immunoblot after tunicamycin challenge alone or in presence of chloroquine. Tunicamycin treatment induced autophagy activation, as indicated by an increase in ATG4B level, and in the conversion of LC3-I to its lipidated form LC3-II (Fig. 1B and C). Chloroquine treatment led to accumulation of LC3B-II in both control and tunicamycin-treated cells, however, LC3B-II accumulation was significantly higher in tunicamycin-treated cells compared to control cells (Fig. 1B and C). Chloroquine also led to an accumulation of p62 in tunicamycin-treated compared to control cells (Fig. 1B and C). These results highlight the notion that autophagic activity is induced after ER stress and alveolar epithelial cell injury.

### Inhibition of autophagy during tunicamycin-induced ER stress promotes apoptosis in mouse lung epithelial cells

**Figure 2:**
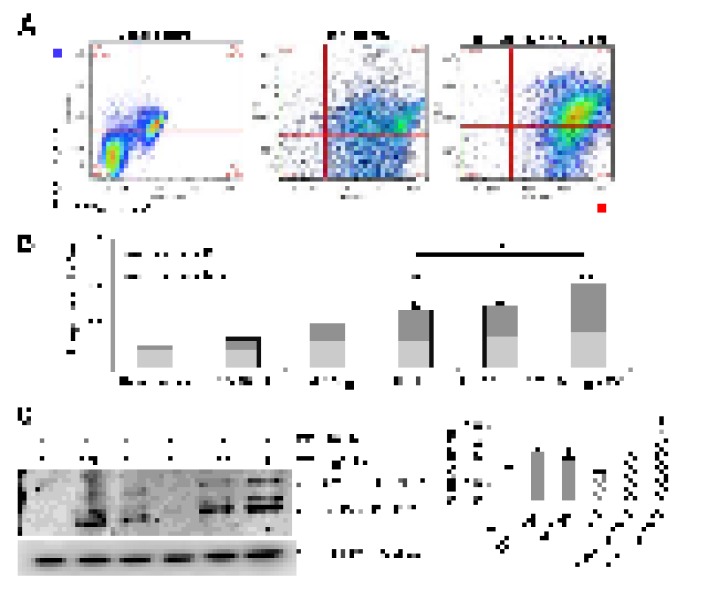
**Disturbance of autophagy rendered mouse alveolar epithelial cells vulnerable to apoptosis after tunicamycin-induced ER stress.** Mouse alveolar epithelial MLE12 cells were treated with vehicle control or 0.5 and 1 μg/ml tunicamycin alone or in combination with chloroquine 10µM for 24h. Apoptosis was determined by flow cytometric analysis of Annexin-V–FITC/ PI-stained cells after 24 h. Representative data (**A**) and cumulative data (**B**) from 3 independent experiments. (**C**) Representative immunoblot of pro- and active CAS3 in mouse alveolar epithelial MLE12 cells treated as indicated. β-tubulin was used as loading control. Densitometry. Protein levels were normalized to vehicle control. Results represent mean ± SD. Statistical significance was determined by one-way ANOVA (* p< 0.05).

Chloroquine-induced inhibition of autophagy during tunicamycin treatment provoked an increased number of apoptotic-like cells with condensed nuclei, round shape and refractive MLE12 cells compared to control, chloroquine alone or tunicamycin alone (Fig. 1A, yellow asterisks in insets). This finding was corroborated

by flow cytometry. As illustrated in Figure 2A and B, after 24 hours of treatment with tunicamycin alone (1 μg/ml), MLE12 cells showed an increased apoptotic rate determined by Annexin V-FITC/PI double staining assay and this effect was significantly enhanced by chloroquine. Chloroquine alone had no significant effect in apoptosis of MLE12 cells. Apoptosis was further examined by immunoblotting, which reveals that chloroquine promotes tunicamycin-induced cell death as observed by an increased expression of cleaved-caspase3 (Fig. 2C). These results suggest that chloroquine could inhibit the cytoprotective effect of autophagy by increasing the cytotoxicity of tunicamycin and promoting apoptosis. Taken together, these findings support the notion that autophagy activity confers protection against ER stress induced injury in lung alveolar epithelial cells.

### Loss of ATG4B is associated with reduced autophagic flux and mild UPR activation in lungs during basal conditions

**Figure 3:**
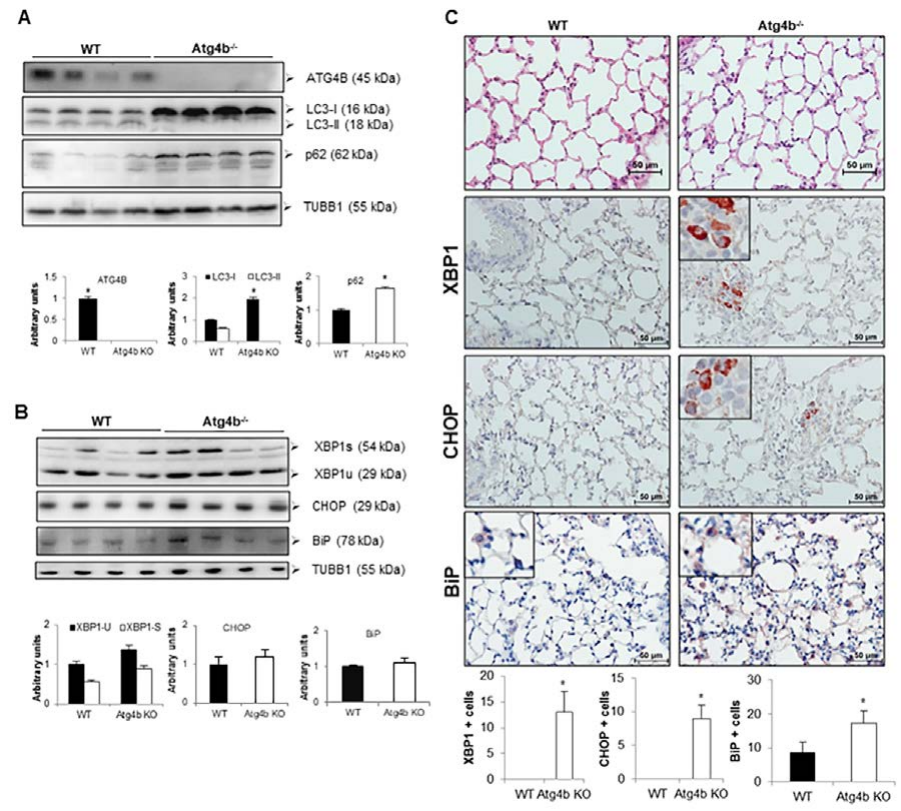
**Loss of autophagy function by Atg4b deficiency resulted in mild ER stress induction.** (**A**) Representative immunoblots of ATG4B, LC3-I/ II, and p62 in lung tissue from WT and Atg4b null mice in basal conditions. (**B**) Representative immunoblots of ER stress biomarkers in lung tissue from WT and Atg4b null mice in basal conditions. β-tubulin was used as loading control. Densitometry analysis (bottom panels). (**C**) Representative photomicrographs of immunohistochemical staining performed with specific primary antibodies against XBP1, CHOP and BiP in lung tissues sections from WT and Atg4b in unchallenged/basal conditions. Positive signal is observed in red. All sections were counterstained with hematoxylin. All insets show 2X larger magnification. Total number of positive stained cells per high power field (40X) in lung tissue sections by quantitative image analysis. Results are shown as mean ± SD. Statistical significance was determined by Student´s t-test (*p < 0.05).

To investigate the association between ER stress and autophagy in vivo, we use our *Atg4b* deficient mouse model. We have previously shown that ATG4B absence does not result in lung developmental defects [15]. However, ATG4B is required for LC3 conjugation and to maintain basal activity of autophagy in lungs. As shown in Figure 3A, we first confirmed by western blot the lacking of ATG4B in lungs from *Atg4b^-/-^* mice. ATG4B deficiency resulted in a significant accumulation of unlipidated LC3 form (LC3-I) and absence of conjugated form or LC3-II as compared to lungs from WT control mice (Fig. 3A). We also found the accumulation of p62, another autophagy-related marker, in the lungs from A*tg4b^-/-^* mice (Fig. 3A). To test whether ATG4B deficiency and autophagy impairment results in spontaneous increase of ER stress, we evaluated by immunoblot and immunohistochemistry the levels and localization of ER stress biomarkers XBP1, CHOP and BiP/Grp78 in lungs form WT and *Atg4b^-/-^* mice under basal conditions. By immunoblot, XBP1, CHOP and BiP protein levels were slightly higher in total lung extracts from *Atg4b^-/-^* compared to WT mice but did not reach statistical significance (Fig. 3B). We observed a strong positive staining for XBP1 and CHOP in a small number of cells from the bronchiolar epithelium in lungs from *Atg4b^-/-^* mice. In sharp contrast, we did not find positive staining for these markers in lungs from WT mice (Fig. 3C). Marginal staining of the chaperone BiP/ Grp78, was found in some alveolar epithelial cells in both *Atg4b^-/-^* and WT lungs, however the number of positive cells was significantly higher in lungs from *Atg4b^-/-^* mice (Fig. 3C). These data demonstrate that in basal conditions, ATG4B disruption and impaired autophagy results in mild UPR activation in lungs from *Atg4b^-/-^* mice.

### ATG4B deficiency enhances susceptibility to tunicamycin-induced ER stress and lung injury

**Figure 4:**
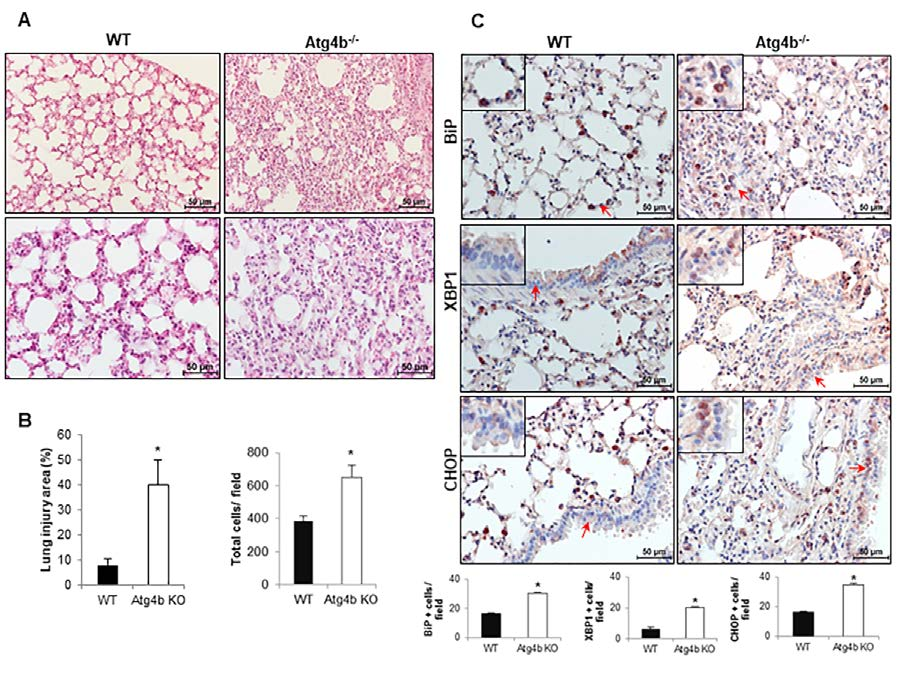
**Exacerbated inflammatory and ER stress response in lungs from Atg4b null mice.** (**A**) Representative light microscopy images of H&E stained lung tissue sections from WT and Atg4b null mice after 3 days post-tunicamycin treatment. (**B**) Lung injury area and total cell number in the lung fields by quantitative image analysis. (**C**) Representative light microscopy images of BiP, XBP1 and CHOP positive stained lung tissue sections from WT and Atg4b null tunicamycin-treated mice at 3 days (top panels). All insets show 2X larger magnification. Total number of positive stained cells per high power field (40X) in lung tissue sections by quantitative image analysis (bottom panels). Results shown represent mean ± SD. Statistical significance was determined by Student’s t test (*p < 0.05).

To determine the role of ATG4B and autophagy in the response to tunicamycin-induced ER stress and its role in lung injury and inflammation, WT and *Atg4b*^-/-^ mice were instilled with tunicamycin (as described in methods), and morphological changes and UPR biomarkers were assessed at 3 days after treatment. Histological analysis revealed that mice from both genotypes exhibited lung inflammation, however *Atg4b^-^*^/-^ mice displayed an enhanced inflammatory response characterized by thickened alveoli, increased cellular infiltration in the alveolar septa and a significantly higher lung injury area relative to WT mice, in which most of the tissue was preserved (Fig. 4A and B). To specifically identify the cellular localization of BiP, CHOP and XBP1 during tunicamycin-induced lung inflammation, we evaluate these proteins by immunohistochemistry. We found BiP positive staining in alveolar epithelial cells and macrophages in lungs from both *Atg4b^-/-^* and WT mice, however the number of BiP positive cells was significantly higher in lungs from *Atg4b^-/-^* mice (Fig. 4C). Tunicamycin-induced ER stress increased the number of XBP1 and CHOP positive cells in WT lungs, where no staining was detected under basal conditions, however, the number of positive cells was significantly higher in lungs from *Atg4b^-/-^* mice. XBP1 and CHOP positive staining was observed in alveolar and bronchiolar epithelial cells and macrophages in lungs from both *Atg4b^-/-^* and WT mice, but remarkably, we observed nuclear staining for XBP1 and CHOP mainly in bronchiolar epithelial only in lungs from *Atg4b^-/-^* mice (Fig. 4C). These data indicate that activation of the UPR after tunicamycin-induced ER stress and the inflammatory response were enhanced in lungs from *Atg4b*^-/-^ mice.

### Tunicamycin induces ER stress but not autophagy in lungs from *Atg4b*^-/-^ mice

**Figure 5:**
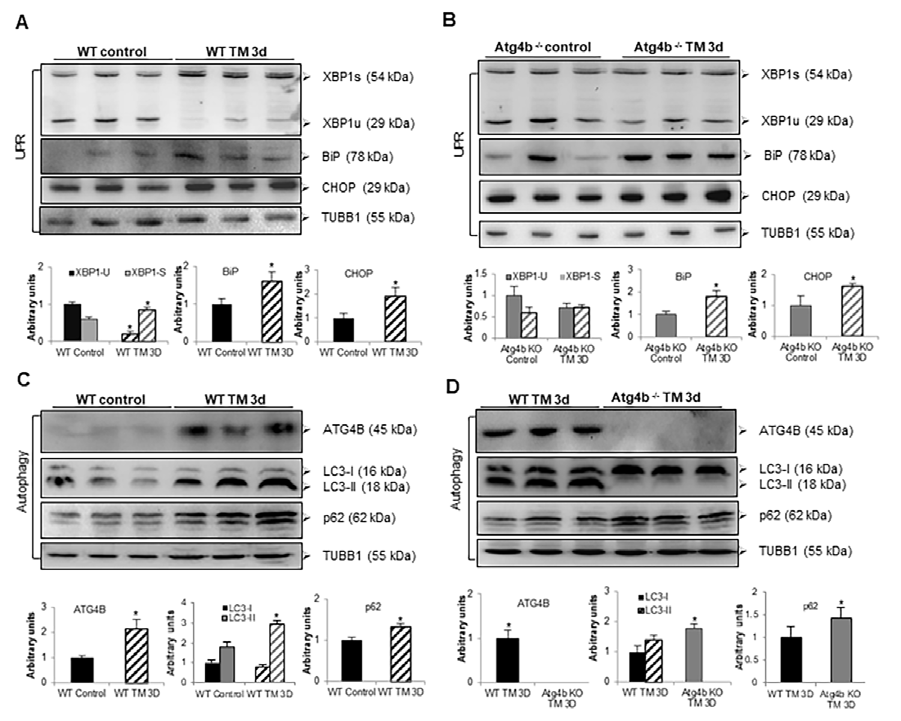
**Tunicamycin treatment induces ER stress but not autophagy in lungs from Atg4b^-/-^ mice.** (**A**) Representative immunoblots of ER stress biomarkers in lung tissue from WT vehicle-control and WT tunicamycin-treated mice at 3 days post-challenge. (**B**) Representative immunoblots of ER stress biomarkers in lung tissue from Atg4b vehicle-control and Atg4b tunicamycin-treated null mice at 3 days post-challenge. (**C**) Representative immunoblots of ATG4B, LC3-I/ II, and p62 in lung tissue from WT vehicle-control and WT tunicamycin-treated mice at 3 days post-challenge. (**D**) Representative immunoblots of ATG4B, LC3-I/ II, and p62 in lung tissue from WT compared to Atg4b null mice after 3 days of tunicamycin treatment. β-tubulin was used as loading control in all experiments. Densitometry analysis (bottom panels). Results are shown as mean ± SD. Statistical significance was determined by Student´s t-test (*p < 0.05).

Previous reports have shown that ER stress can stimulate autophagy as a cytoprotective mechanism that promotes cell survival [10,11,21]. To assess whether tunicamycin treatment leads UPR and autophagy activation in lung, autophagic activity and the levels of ER stress biomarkers were evaluated at 3 days after TM instillation. It is well known that XBP1 protein exists as 29-kDa transcriptionally inactive unspliced (XBP1u) and 56-kDa transcriptionally active spliced (XBP1s) isoforms [21–23]. We observed that tunicamycin treatment results in processing of XBP1**-**u into its active XBP1-s form in lungs from WT compared to vehicle control mice. However, no processing of XBP1-u was noticed in lungs from TM-treated compared to vehicle-control *Atg4b^-/-^* mice (Fig. 5A and B). Tunicamycin instillation also elicited an increased expression of BiP in lungs from both *Atg4b^-/-^* and WT mice. Increased expression of CHOP was also noticed in lungs from both TM-treated *Atg4b^-/-^* and WT mice, relative to lungs from vehicle controls, but it was far less than BiP (Fig. 5A and B). Lung tissue extracts from TM-treated WT mice showed a significant increase of the enzyme ATG4B, and also a marked accumulation of LC3-II form and p62, compared to lung tissue from vehicle WT controls, indicating autophagy activation (Fig. 5C). Conversely, in lungs from TM-treated *Atg4b^-/-^* mice, a strong accumulation of LC3-I and complete absence of LC3-II form were observed, which indicates that these mice are unable to activate autophagy in response to tunicamycin-induced ER stress (Fig. 5D). In summary, these results support the notion that concomitantly with tunicamycin-induced ER stress, autophagy and ATG4B expression are induced in lungs from WT mice. However, due to ATG4B disruption, autophagy is not activated and XBP1u to XBP1s conversion is impaired in lungs from *Atg4b^-/-^* mice despite ER stress conditions.

### Apoptosis is increased in *Atg4b*^-/-^ lungs after tunicamycin-induced ER stress

**Figure 6:**
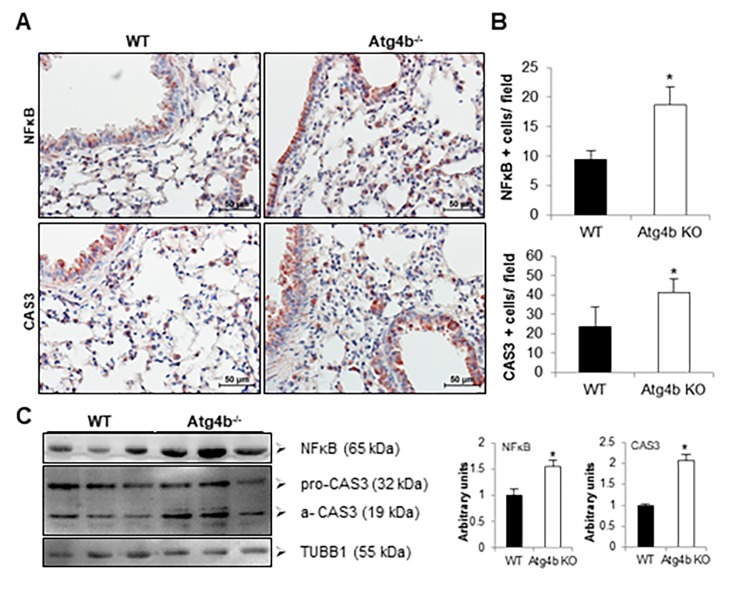
**Atg4b deficiency resulted in increased NFκB and CAS3 expression in mouse lung at 3 days after tunicamycin administration.** (**A**) Representative photomicrographs of immunohistochemical staining performed with specific primary antibodies against NFκB and CAS3 in lung tissues sections from WT and Atg4b tunicamycin-treated mice at 3 days post-instillation. Positive signal is observed in red. All sections were counterstained with hematoxylin. (**B**) Total number of positive stained cells per high power field (40X) in lung tissue sections by quantitative image analysis. (**C**) Representative immunoblots of NFκB and CAS3 in lung tissue from WT and Atg4b null mice at 3 days post-tunicamycin instillation (left panel) and densitometry analysis (right panel). Results are shown as mean ± SD. Statistical significance was determined by Student´s t-test (*p < 0.05).

We have demonstrated in vitro that ER stress-induced autophagy provides cytoprotection to epithelial cells against tunicamycin. To identify cells undergoing apoptosis in the lungs from WT and mutant mice after 3 days post-tunicamycin instillation, immunohistochemistry for caspase-3 was performed. CAS3 positive staining was mainly observed in bronchial and alveolar epithelial cells and also in macrophages in lungs from both *Atg4b^-/-^* and WT mice (Fig. 6A and B). However, the number of CAS3 positive cells was significantly higher in *Atg4b^-/-^* mice. This finding was confirmed by immunoblot in lung tissue extracts, where we observed a significant increase in active CAS3 protein level in *Atg4b^-/-^* mice, compared to WT lungs (Fig. 6C). Additionally, we also evaluate the level of NFκB/RelA (p65), a key transcription factor that activates inflammation, and may promote apoptosis in response to cellular stress [24,25]. We observed an increased number of NFκB positive cells, as well as significantly higher levels of NFκB protein in lung tissue extracts after 3 days post-tunicamycin instillation in lungs from *Atg4b^-/-^* compared to WT mice (Fig. 6A, B and C). In summary, our data reveal that ATG4B deficiency and impaired autophagy, results in an exacerbated and altered UPR response that could culminate in increased apoptosis and enhanced inflammatory response.

### Tunicamycin-induced ER stress promotes lung fibrosis in *Atg4b* deficient but not in WT mice

**Figure 7:**
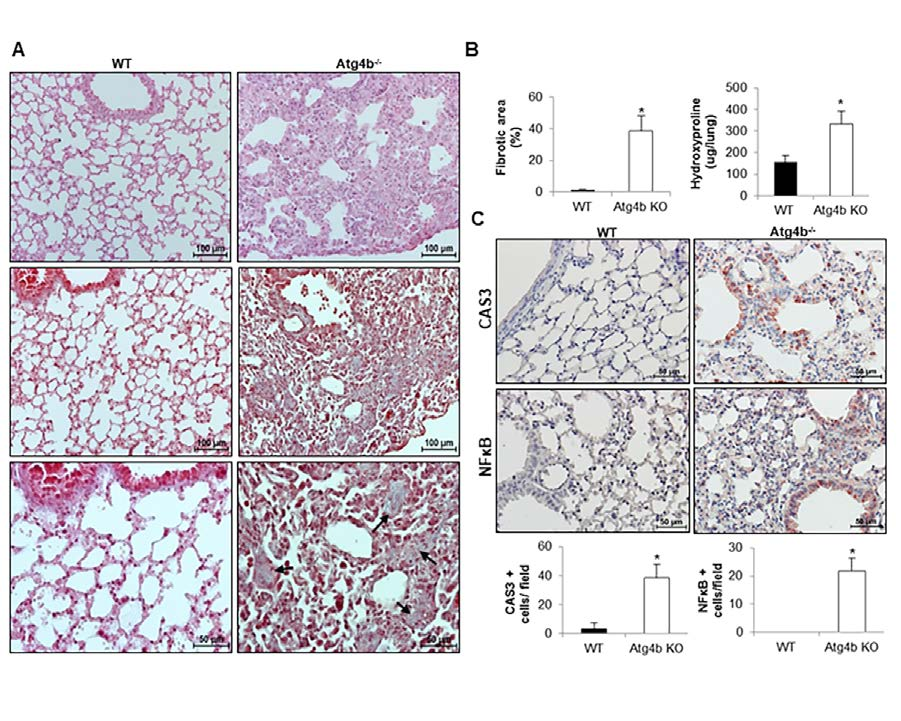
**Atg4b deficiency and impaired autophagy sensitizes mice to epithelial apoptosis and lung fibrosis.** (**A**) Representative light microscopy images of H&E and Masson’s trichrome stained lung tissue sections from WT and Atg4b null mice at 21 days post-tunicamycin instillation. (**B**) Fibrosis extent percentage of damaged tissue area in the lung fields by quantitative image analysis and hydroxyproline content in lungs from tunicamycin-treated WT and Atg4b null mice. (**C**) Representative light microscopy images of NFκB and CAS3 positive stained lung tissue sections from WT and Atg4b null tunicamycin-treated mice at 21days (top panels). Total number of positive stained cells per high power field (40X) in lung tissue sections by quantitative image analysis (bottom panels). Results shown represent mean ± SD. Statistical significance was determined by Student’s t test (*p < 0.05).

A growing body of evidence suggests that ER stress promotes fibrotic remodeling through activation of pro-apoptotic pathways and promotion of inflammatory response [6,8,9]. To determine if the inflammatory process observed in *Atg4b*^-/-^ mice after tunicamycin-induced ER stress could progress to pulmonary fibrosis, WT and *Atg4b*^-/-^ mice were treated with a single intratracheal dose of tunicamycin or vehicle control and lungs were harvested at 21 days post-instillation (fibrotic phase). Histological examination of lung sections stained with hematoxylin and eosin, and Masson’s trichrome staining for collagen deposition, underscored that WT mice did not developed pulmonary fibrosis. Thus, lungs from TM- treated WT mice showed a morphology similar to vehicle control lungs, and collagen was identified only in areas surrounding large vessels and airways. Conversely, lungs from TM-treated *Atg4b*^-/-^ mice exhibited thickened alveolar septa and intra-alveolar and interstitial collagen deposition (Figure 7A, black arrows). Morphometric analysis of the fibrotic areas as well as the quantification of hydroxyproline concentration confirmed that collagen deposit was significantly higher in the lungs from TM-treated *Atg4b*^-/-^ mice compared to TM-treated WT after 21 days (Figure 7B). Additionally, an increased number of bronchiolar and alveolar epithelial cells stained for CAS3 and NFκB were found in TM-treated *Atg4b*^-/-^ lungs at 21 days post-tunicamycin treatment, while few CAS3 positive signal and no staining for NFκB was found in TM-treated WT lungs (Figure 7C). According to the data shown so far, our findings suggest that autophagy activation in WT lungs could restore defective proteostasis upon detection of unfolded protein in the ER lumen, limiting epithelial apoptosis, reducing the severity of inflammatory response and preventing pulmonary fibrosis development. In conclusion, our findings indicate that ATG4B deficiency, and impaired autophagy could compromise lung cells to reach proteostasis and enhances the susceptibility of epithelial cells to ER stress-induced pro-apoptotic and pro-fibrotic stimuli.

## DISCUSSION

Aging can significantly impair ER function through reduction in chaperone production, defects in protein folding, and inefficient degradation of proteins via proteasome or autophagy [27,28]. ER stress markers are common in the lungs of patients with both familial and sporadic IPF and it has been suggested that it is involved in the pathogenesis [6,7]. Along with ER stress and UPR activation, a reduced rate of autophagy has been found in IPF lungs [12,13]. Mutations in surfactant protein C, viral infections, reactive oxygen species (ROS) generated by cigarette smoke or inhaled particulates and abnormal or reduced clearance of misfolded and damaged proteins have been identified as ER stress inducing factors in this disease [27,28]. Additionally, previous studies have shown that inflamed airway epithelia alter ER homeostasis and trigger the UPR. It has been demonstrated that mucin production by distal airway cells is mediated by IRE1β and XBP1 activation under physiological conditions, but during airway epithelial inflammation, IRE1 activation resulted in the expansion of ER Ca^2+^ stores in ciliated cells, which provides a mechanism for amplification of Ca^2+^-dependent cytokine secretion and mucin production [29]. Recently, a single nucleotide polymorphism (SNP) in the MUC5B gene (rs35705950) has been strongly associated with familial and sporadic IPF. However, the relationship between ER stress and mucus secretion in IPF lungs is still unknown [30–32].

Previous studies have shown the presence of ER stress, UPR activation and epithelial to mesenchymal transition in alveolar epithelial cells after TM administration or mutant L188Q SFTPC expression [9,33]. Our study in mouse MLE12 AEC confirmed that TM treatment increased expression of UPR biomarkers such as BiP, XPB1s and CHOP, but reveals for the first time that UPR activation in lung epithelial cells is accompanied by increased ATG4B expression and autophagy activation. Autophagy has been associated with both pro-apoptotic and pro-survival functions [34,35]. Thus, we evaluated the regulatory effects of autophagy on ER stress induced by tunicamycin, using chloroquine to block autophagosome fusion with lysosome and to inhibit autophagosome cargo degradation. We found that MLE12 cells treated with chloroquine and tunicamycin where more sensitive to cell death suggesting that a proper autophagic activation is important for relieve proteostatic stress and promote cell survival.

We and others have previously demonstrated that ATG4B is a cysteine protease required for autophagy [14,16–18]. Here, we addressed the role of autophagy in regulating ER stress in vivo, taking advantage of our *Atg4b* KO model and analyzed how defective autophagy could influence UPR and impact on cell death, inflammation and fibrotic remodeling in lung.

The UPR is initiated by the activation of three specialized stress sensors, including IRE1α (inositol-requiring transmembrane kinase/endonuclease), PERK (PKR-like ER kinase) and ATF6 (activating transcription factor 6). UPR downstream effectors include BiP, CHOP and XBP1 [27,36,37]. Different studies have shown that deficiency in UPR effectors such as CHOP and XBP1 induce an increase in Atg proteins and autophagic activity to exert a cytoprotective effect [10]. Accordingly, strong evidence indicates that, loss of autophagy function by conditional deficiency in Atg7 in T lymphocytes or Atg5 in B lymphocytes/plasma cells resulted in expanded ER and increased ER stress signaling, supporting a role for autophagy in ER homeostasis [38,39]. Moreover, knockout of Atg5 or Atg7 in pancreatic acinar cells produces ER dilation, stress, cell death and inflammation [40,41]. Therefore, to evaluate if ATG4B deficiency and autophagy impairment activates UPR, we evaluated the expression levels and cell localization of BiP, XBP1 and CHOP in lungs from *Atg4b*^-/-^ and WT mice under basal conditions. We found that ATG4B deficiency and concomitant reduced autophagic activity stimulated a mild UPR with few cells positive for XBP1 and CHOP in mutant mice, while in WT lungs these markers were completely absent. BiP was expressed at basal levels in alveolar epithelial cells in WT lungs, indicating that this chaperone could be a gatekeeper of ER homeostasis; however, an increased number of BiP positive cells were found in *Atg4b* defective mutant lungs. This mild UPR activation could be a compensatory phenomenon to maintain cellular homeostasis in *Atg4b*^-/-^ lungs.

Then, we addressed whether autophagy-defective mutant mice were more sensitive than WT to lung ER stress induced by intratracheal instillation of tunicamycin. We found that exposure to tunicamycin, resulted in widespread bronchiolar and alveolar damage in both WT and *Atg4b*^-/-^ lungs. However higher Cas3 activation and increased expression of NFKB, BiP, XBP1 and CHOP was observed in bronchiolar and alveolar epithelium from *Atg4b*^-/-^ compared to WT lungs, confirming that autophagy contributes to ER stress attenuation.

Recently, a link between ER stress elevation and high mucin biosynthesis has been found in bronchial epithelium. It has been found that mucin production is mediated by IRE1β-dependent XBP1 activation in primary cultures of human bronchial epithelia (HBE). In vivo, Clara cells in lungs from *Ire1β*^−/−^ mice produce low levels of MUC5B after ovalbumin challenge [29]. On the other hand, subjects with IPF had significantly higher levels of MUC5B and this protein was localized in bronchial secretory columnar cells and in larger proximal bronchioles. It has been speculated that high MUC5B levels in the respiratory bronchioles may impaired alveolar repair by interfering with the interaction between the type II alveolar epithelial cells and the underlying matrix or by interfering with the surface-tension properties of surfactant leading to failure in re-epithelialization and aberrant wound healing [30,32]. However, co-localization of this protein with ER stress biomarkers in IPF lungs has not been reported so far.

Regarding to the alveolar epithelium and ER stress, it has been previously reported that transgenic expression of the mutant L188QSFTPC in AEC or direct intratracheal instillation of tunicamicyn into the lung, resulted in ER stress but not in AEC apoptosis or pulmonary fibrosis [6]. However, induction of ER stress combined with a low dose bleomycin resulted in a severe fibrotic response compared with bleomycin alone [6,9]. These results suggest that ER stress in AEC, is not sufficient to induce lung fibrosis but sensitize animals to develop fibrosis following epithelial injury [6,27]. In our study however, we found intra-alveolar and interstitial collagen deposition in lungs from tunicamycin-challenged *Atg4b*^-/-^ mice at 21 days post-treatment, while WT mice were protected, suggesting that ER stress alone is sufficient to promote lung fibrosis when autophagic activity is compromised. Several lines of evidence support that apoptosis of epithelial cells is a key event initiating and promoting pulmonary fibrosis [1–3]. Remarkably, we observed that bronchiolar and alveolar epithelium were still very reactive for NFKB and Cas3 staining after 21 days post-tunicamycin challenge in *Atg4b*^-/-^ lungs, while positive staining was barely detected in WT lungs. These findings suggest that in the WT lung epithelial cell turns on autophagy as a pro-survival mechanism and ER stress is resolved. By contrast, an abnormal autophagic activity in *Atg4b*^-/-^ lungs fails to protect the cells from stress, and protein accumulation and ER stress could be sustained, leading to a defective repair that may itself contribute to epithelial apoptosis [9,14,19,20]. In conclusion, our study shows that *Atg4b*-deficient lung epithelial cells appear to be particularly sensitive to apoptosis following ER-stress lung injury, which combined with autophagy deficiency results in synergistic proinflammatory and pro-fibrotic activities. Our findings confirm that autophagy is part of an adaptive response to protect cells against ER stress and it is closely engaged with the UPR pathway. We propose that our *Atg4b* deficient model mimics a pathogenic scenario in which lung fibrosis is favored by aging associated drivers such as abnormal autophagy and ER stress. Future work will expand our understanding about the crosstalk between ER stress, UPR and autophagy during pulmonary fibrosis.

## MATERIALS AND METHODS

### Culture and treatment of MLE-12 cells

Mouse lung epithelial cell line MLE-12 was purchased from American Type Culture Collection (ATCC, CRL2110). MLE 12 cells were cultured in a CO_2_ incubator (5% CO_2_–95% air) at 37°C with HITES medium (F-12/DMEM, Life Technologies, Inc., Gaithersburg, MD), supplemented with 0.01% hydrocortisone, 1% insulin-transferrin-sodium selenite (Sigma-Aldrich), 0.01% β-estradiol, 100 units/ml penicillin, 100 μg/ml streptomycin, 1% L-glutamine, 1% HEPES, 0.01% β-estradiol (Sigma-Aldrich, E2257), 0.01% hydrocortisone and 2% fetal bovine serum. MLE 12 cells were exposed to 0.5 or 1µg/ml of tunicamycin (Sigma-Aldrich, T7765) with or without chloroquine 10 µM (Sigma-Aldrich, C6628) for 24 h.

### Flow cytometry

Cells were grown to 75% confluence in 12-well plates. After stimulation with tunicamycin and chloroquine, cells were harvested and double stained with annexin-V and PI. We used FITC Annexin V Apoptosis Detection Kit with PI (BioLegend, San Diego, CA) to quantitate the early and late apoptosis. The protocol is described briefly according to the manufacturer’s protocol. MLE-12 cells were treated with indicated chemicals for 24 h, and then were trypsinized and washed twice with cold BioLegend cell staining buffer. The cell suspension was transferred to a 5 ml test tube, and then 5 μl of FITC Annexin V and 5 μl of Propidium Iodide (PI) solution were added. The cells were gently vortexed and incubated for 15 min at room temperature (25 °C) in the dark. Four hundred μl of Annexin V binding buffer was added to each tube and finally were analyzed using a BD FACS Calibur flow cytometer (BD Biosciences). Analysis and visualization of the flow cytometry data were performed using FlowJo software (Tree Star, Inc., Ashland, OR). Three independent experiments were performed.

### Animals and ER stress induction

The generation of *Atg4b*^−/−^ mice has been previously described (14). Mice genotypes were determined by PCR analysis of tail DNA. Mice were bred under specific pathogen-free conditions. All experiments were performed with 8- to 10-wk-old mice and were approved by the Committee on Animal Experimentation of the National Institute of Respiratory Diseases of Mexico (INER). Lung injury was induced by intratracheal instillation of tunicamycin (Sigma-Aldrich, T7765) at a single dose of 10 µg in 50 μl of vehicle (2 mg/mL). Control groups received the same volume of vehicle. Mice were sacrificed at 3 and 21 days after tunicamycin or vehicle treatment. For tissue harvesting, the lungs were perfused with sterile saline from right to left ventricle of the heart. Lungs were removed for fixation overnight in paraformaldehyde or snap frozen in liquid nitrogen followed by storage at –80°C.

### Morphology

Lungs were removed and fixed by inflation with 4% paraformaldehyde in 1× PBS at continuous pressure of 25 cm H_2_O and were embedded in paraffin. Sections were stained with hematoxylin-eosin and Masson trichrome stain, and were scored blindly for severity and extent of lung lesions. To measure focal areas of fibrosis in the interstitium as well as in the intra-alveolar spaces in the total lung area, 6 fields were randomly chosen at × 400 magnification in each slide and ROI sizes were manually calculated using NIS-Elements software. Positive cells quantification was accomplished post-acquisition using NIS-Elements Nikon software.

### Immunohistochemistry

The tissue sections were deparaffinized and were then rehydrated and blocked with 3% H2O2 in methanol followed by antigen retrieval in a microwave in 10 mM citrate buffer, pH 6.0. Tissue sections were treated with universal blocking solution (BioGenex, HK085–5K) for 10 min, and then incubated overnight at 4°C with the following primary antibodies: anti-XBP1 (Santa Cruz Biotechnology), anti-CHOP (Sigma-Aldrich,), anti-BiP (Sigma-Aldrich,), anti-pro/active-CASP3/caspase 3 (Novus Biologicals) and anti-NFκB (Abcam). A secondary biotinylated anti-immunoglobulin followed by horseradish peroxidase-conjugated streptavidin (BioGenex, HK330–5K) was used according to the manufacturer's instructions. 3-amino-9-ethyl-carbazole (BioGenex, HK092–5K) in acetate buffer containing 0.05% H2O2 was used as the substrate. The sections were counterstained with hematoxylin. The primary antibody was replaced by nonimmune serum for negative control slides. Image analysis and cell counting was performed using the Nikon software, Nis Elements 3.0.

Positive signals were distinguished from cytoplasmic staining and were quantified using automated image analysis from different fields at 40X magnification. All photomicrographs were taken with the same settings for exposure time and light. The results are represented in bar graphs as the percentage of positive cells per field.

### Immunoblotting

Lung tissue or MLE12 cells were homogenized in a 20 mM Tris buffer pH 7.4, containing 150 mM NaCl, 1% Triton X-100 (Sigma-Aldrich, T8787), 10 mM EDTA and UltraCruz Protease Inhibitor Cocktail (Santa Cruz Biotechnology, sc-29131). Tissue and cell extracts were centrifuged at 15,000 × g at 4°C and supernatant fractions were collected. Protein concentration was quantified by bicinchoninic acid technique (BCA protein assay kit, Pierce Biotechnology, 23225). A total of 25 µg of protein was loaded on either 8% or 13% SDS-polyacrylamide gels. After electrophoresis, gels were electrotransferred onto polyvinylidenedifluoride membranes (PVDF, Millipore, IPV H00010), and then membranes were blocked with 5% nonfat dried milk in TBS-T (Tris-buffered saline with 0.05% Tween 20 [Sigma-Aldrich, P9416]) and incubated overnight at 4°C with the following primary antibodies diluted in antibody diluent (Thermo-Fisher Scientific, 003118): anti-ATG4B (Sigma-Aldrich, A2981), anti-SQSTM1 (Sigma-Aldrich, P0068), anti-LC3B (Sigma-Aldrich, L7543), anti-TUBB4 (Santa Cruz Biotechnology, sc-9104), anti-β-tubulin (Santa Cruz Biotechnology, sc-47778), anti-XBP1 (Santa-Cruz, sc-7160), anti-CHOP (Sigma-Aldrich, NB600-1335), anti-BiP (Sigma-Aldrich, G8918), anti-pro/active-CASP3/caspase 3 (Novus Biologicals) or anti-NFκB (Abcam) . After 3 washes with TBS-T, membranes were incubated with the corresponding secondary antibody at 1:3000 dilution in 1.5% milk in TBS-T, and developed with Immobilon Western Chemiluminescent HRP substrate (Millipore, WBKLS0500).

### Statistics

All experimental data are reported as mean ± SD. Statistical analyses were performed by one-way ANOVA and Student t test using Graphpad Prism Software Version 4.0 (Graphpad Software Inc., San Diego CA) and P values lower than 0.05 were considered significant.
